# Multiple gene variations contributed to congenital heart disease via GATA family transcriptional regulation

**DOI:** 10.1186/s12967-017-1173-0

**Published:** 2017-04-03

**Authors:** Yanyan Qian, Deyong Xiao, Xiao Guo, Hongbo Chen, Lili Hao, Xiaojing Ma, Guoying Huang, Duan Ma, Huijun Wang

**Affiliations:** 1 Key Laboratory of Metabolism and Molecular Medicine, Ministry of Education, Department of Biochemistry and Molecular Biology, Collaborative Innovation Center of Genetics and Development, Institutes of Biomedical Sciences, School of Basic Medical Sciences, Fudan University, Shanghai, 20032 China; 2 Shanghai Key Lab of Birth Defect, Children’s Hospital of Fudan University, Shanghai, 201102 China; 3 Pediatric Heart Center, Children’s Hospital of Fudan University, Shanghai, 201102 China; 4 Research Center for Birth Defects, School of Basic Medical Sciences, Fudan University, 130 Dongan Road, Shanghai, 200030 China; 5 Molecular Genetics Laboratory, Children’s Hospital of Fudan University, 399 Wanyuan Road, Shanghai, 201102 China

**Keywords:** Congenital heart disease (CHD), Next-generation sequencing, Functional study, Multigene disease, Variants

## Abstract

**Background:**

Congenital heart disease (CHD) is a common birth defect, and most cases occur sporadically. Mutations in key genes that are responsible for cardiac development could contribute to CHD. To date, the genetic causes of CHD remain largely unknown.

**Methods:**

In this study, twenty-nine candidate genes in CHD were sequenced in 106 patients with Tetralogy of Fallot (TOF) using target exome sequencing (TES). The co-immunoprecipitation (CO-IP) and luciferase reporter gene assays were performed in HEK293T cells, and wild-type and mutant mRNA of *ZFPM2* were microinjected into zebrafish embryos.

**Results:**

Rare variants in key cardiac transcriptional factors and *JAG1* were identified in the patients. Four patients carried multiple gene variants. The novel E1148K variant was located at the eighth Zinc-finger domain of FOG2 protein. The CO-IP assays in the HEK293T cells revealed that the variant significantly damaged the interaction between ZFPM2/FOG2 and GATA4. The luciferase reporter gene assays revealed that the E1148K mutant ZFPM2 protein displayed a significantly greater inhibition of the transcriptional activation of GATA4 than the wild-type protein. The wild-type mRNA and the E1148K mutant mRNA of ZFPM2 were injected into zebrafish embryos. At 48 hpf, in the mutant mRNA injection group, the number of embryos with an abnormal cardiac chamber structure and a loss of left–right asymmetry was increased. By 72 hpf, the defects in the chamber and left–right asymmetry became obvious.

**Conclusions:**

We performed TES in sporadic TOF patients and identified rare variants in candidate genes in CHD. We first validated the E1148 K variant in ZFPM2, which is likely involved in the pathogenesis of CHD via GATA4. Moreover, our results suggest that TES could be a useful tool for discovering sequence variants in CHD patients.

**Electronic supplementary material:**

The online version of this article (doi:10.1186/s12967-017-1173-0) contains supplementary material, which is available to authorized users.

## Background

Congenital heart disease (CHD), which occurs in approximately 1% of newborns, is the most common birth defect worldwide [1]. However, the etiology of CHD remains largely unknown. Currently, the prevalence of CHD in China has reached approximately 26.6 per 1000 newborns [2]. Cyanotic CHD was a type of a severe congenital disease that threatens newborns’ survival and quality of life, and Tetralogy of Fallot (TOF) is the most prevalent type [3]. TOF was characterized by the tetrad of over-riding aorta, ventricular septal defect, pulmonary valve stenosis and right ventricular hypertrophy. It is widely accepted that CHD is a multigenic inheritance disease. Cardiac development involves numerous elaborate regulations. Transcription factors [4], developmental pathway molecules [5, 6] and epigenetic regulators [7] are essential for normal cardiac development. Mutations in these genes may disturb normal signaling transduction, which could lead to CHD [8–10].

NKX, GATA and T-box family members constitute the core regulatory network that is responsible for normal cardiac morphogenesis and are causative genes in CHDs [11, 12]. The GATA family members were characterized by two zinc finger domains and transcriptional activation domains (TADs). The two TAD domains of GATA4 are located at the N- and C-terminal, respectively, different from GATA5 or GATA6, which located in N-terminal [13–15]. Mutations in *GATA4*, *GATA5* and *GATA6* were identified in patients with CHD [16–18]. The *ZFPM2* gene encodes the FOG2 protein. FOG2 is a transcription regulator of the GATA family members that can not only bind with GATA but can also recruit the nucleosome remodeling and deacetylation (NuRD) complex to moderate the GATA-mediated gene regulation [19, 20]. Genetic analysis revealed that mutations in *ZFPM2* disrupted the interaction with GATA4 or the NuRD complex, leading to CHD [19, 21–23]. During cardiogenesis, the Notch signaling pathway plays significant roles in cell fate specification and tissue patterning [24]. Notch signaling could also regulate the GATA-dependent cardiac gene expression [25]. Mutations in elements along this pathway result in cardiac malformation [26]. *JAG1* is expressed in the mesocardium, pulmonary artery and aorta during embryogenesis [27]. The conditional knock-out of Jag1 in the cardiac neural crest or endocardium led to cardiac defects [28, 29].

In this study, we designed a gene panel, which contained twenty-nine candidate genes in CHD, including the key cardiac transcription factors and other cardiogenesis-related genes. The coding regions of these genes were analyzed in 106 patients with TOF using target exome sequencing (TES) methods. Rare variants in the cardiac transcription factors and Notch signaling pathway elements were identified. Additionally, a novel variant in *ZFPM2* was selected for a functional study. Our results suggest that TES could be a useful tool for discovering novel pathogenic variants in causative genes in sporadic CHD.

## Methods

### Study subjects and samples

The 106 non-syndromic patients with TOF, who were enrolled at the Children’s Hospital of Fudan University, were aged between 1 and 214 months. All patients were diagnosed by echocardiography. There were 65 boys and 41 girls, and the average age is 18.6 months. Among the patients, there were 40 cases with only simplex TOF, which accounted for approximately 37.7%. There were 38 patients with TOF with PFO or/and PDA, which accounted for approximately 35.8%. There were 8 patients who suffered from TOF with ASD or AVSD, and 2 patients had TOF with LSVC. The general clinical characteristics of the 106 patients are summarized in Table 1 and Additional file 1: Table S1. All peripheral venous blood samples from the patients used in this study were collected with the appropriate informed consent and the approval of the ethics committees of the Children’s Hospital, Fudan University (2014-107). All genomic DNA samples were extracted from whole blood using the QIAamp DNA Blood Mini Kit (Qiagen, Germany) following the manufacturer’s protocol. The quality and quantity of the DNA samples were measured using the NanoDrop 2000 spectrophotometer (Thermo Fisher Scientific, USA).

**Table 1:** Clinical characteristics of the patients

Subgroup	Number	Percentage/range
Male	65	61%
Female	41	39%
Age at diagnosis (months)	18.6	1–216
Diagnosis types of TOF		
TOF	40	38%
TOF + ASD	6	6%
TOF + AVSD	2	1%
TOF + LSVC	2	1%
TOF + PDA	1	1%
TOF + PFO	29	28%
TOF + PDA + PFO	8	8%
TOF + Others	18	17%

*TOF* Tetralogy of Fallot, *ASD* atrial septum defect, *AVSD* atrioventricular septum defect, *LSVC* left superior vena cava, *PDA* patent ductus arteriosus, *PFO* patent foramen ovale

### CHD panel design

Twenty-nine genes were included in this CHD panel, including key cardiac transcription factors, and structural proteins. All genes were reported to have had mutations in human CHD patients and were deposited in the human genome mutation database (HGMD: http://www.biobase-international.com/) (Additional file 2: Table S2). The primers were designed online using life technology (https://www.ampliseq.com/). The Ampliseq primers were designed to cover all exons and at least 10 bp of all intron/splice sites.

### Candidate genes sequencing

The ion Torrent PGM™ platform was used for the sequencing. The Ampliseq panel library was derived from multiple PCR reactions that were conducted using the Ion AmpliSeq™ HiFi Mix and Ion AmpliSeq™ Primer Pool, in addition to the digestion of the primer sequences (Ion AmpliSeq™ Library Kits 2.0), followed by adaptor and barcode ligation (Ion Xpress Barcode Adapters Kit, Life Technologies, USA). The libraries were quantified using real-time PCR by the IonTaq Assay (Ion Library Quantification Kit, Life Technologies) and diluted to 100 pM. Different patients were distinguished by the barcode ligation (Ion Xpress Barcode Adapters Kit, Life Technologies, USA). Therefore, according to the manufacturers of the kits, we could also use Amplicon to add other candidate genes or any gap to the same sample using the same barcode in one run. We used the Ion OneTouch™ system (Life Technologies, USA) to clonally amplify the pooled barcoded libraries of the Ion Sphere™ particles. Torrent Suite™ software was used to compare the base calls. We pooled 8–12 cases of barcoded sample libraries on one 316™ or 316v2™ chip for sequencing. In addition, the average sequence depth of each case was greater than 400X, with 95% over 20X coverage.

### In silico analysis

Torrent Suite™ software was used to compare the base calls. Then, the licensed NextGENe software (Softgenetics, USA) was used to complete the data analysis. The VCF collected from torrent suite were also annotated by ANNOVAR and VEP. All SNVs were compared to 1000 genomes, ExAC and our laboratory’s internal databases. We have data for more than 3500 Chinese non-cardiac disease cases. In addition, the risk of single-nucleotide variations (SNVs) was predicted by the SIFT (http://sift.jcvi.org/), polyphen 2 (http://genetics.bwh.harvard.edu/pph2/) and MutationTaster (http://www.mutationtaster.org/) software. The UCSC website was used to compare the human wild-type proteins to orthologs from different species.

### Sanger sequencing

The candidate SNVs were amplified by the PCR primers from the genomic DNA and sequenced by Sanger sequencing. The forward and reverse primers were designed by the program Primer 3.0 online. The PCR products were sequenced using the BigDye Terminator v3.1 Cycle Sequencing Kit (Applied Biosystems, USA) with an ABI 3730 Genetic Analyzer. The sequence data were analyzed by the Mutation Surveyor Software (Softgenetics, USA).

### Co-immunoprecipitation

The coding regions of human *ZFPM2* and *GATA4* were amplified and tagged with a Flag-tag or HA-tag using the PCR methods and were then cloned into the mammalian expression vector pCDH (Clontech, USA). The mutant E1148 K of *ZFPM2* was created using the Toyobo KOD-Plus Mutagenesis Kit (Code No SMK-101) according to the manufacturer’s instructions. HEK293T cells were transiently co-transfected with the *GATA4* and wild-type or mutant of *ZFPM2* plasmids using the Viafect transfection agent (Promega, USA). After 48 h of transfection, the cells were collected for the experiments. The cell lysates were incubated with the HA affinity gel (Biotool, USA) overnight at 4 °C. The interactions between GATA4 and the wild-type or mutant ZFPM2 protein were detected by western blotting. The antibodies used for the western blotting were anti-GATA4 (Genetex, USA), anti-FOG2 (Santa Cruz, USA), anti-Flag (Abmart, China), and anti-HA (Abmart, China). Three independent experiments were performed. The ImageJ software was used for the semi-quantitative analysis to acquire a digital number for the analysis. GraphPad 5.0 was used for the statistical analysis.

### Reporter gene assay

The 2600-bp 5′-flanking region of the atrial natriuretic factor (ANF) gene was amplified from normal human DNA using the following specific primers: ANF-F: 5′ CTAGCTAGCAGTGACCTCCATATTA-3′, ANF-R: 5′ CCGCTCGAGTGCTGGCGTCGTCAAG-3′. Then, the sequence was cloned into the pGL3 basic vector (Promega, Madison, WI). In the co-transfection experiments, 500 ng of the pCDNA3-hGATA4 plasmid, 500 ng of the wild-type pCDH-hZFPM2 plasmid, 500 ng of the mutant pCDH-hZFPM2 plasmid, and 500 ng of the ANF construct were used. pRLRenilla (Promega, Madison, WI) was used as a normalized plasmid. HEK293T cells were transfected with the plasmids using the Viafect transfection agent (Promega, Madison, WI). After 48 h of transfection, the luciferase and Renilla luciferase activities were measured using the Dual-Glo luciferase assay system (Promega, Madison, WI). The activity of the ANF promoter is presented as the fold activation of firefly luciferase relative to that of Renilla luciferase. The experiments were performed in triplicate.

### Zebrafish experiments

The wild-type zebrafish embryos of the Tu and transgenic cmlc2 were as follows: eGFP (cardiac myosin light chain 2: eGFP reporter) strains were used in this study. Adult zebrafish were reared under standard aquaculture conditions at 28.5 °C on a 14/10 h light/dark cycle. The embryos were collected after the group mating and kept in an embryo medium (17 mM NaCl, 0.2 mM KCl, 0.18 mM Ca (NO_3_)_2_, 0.12 mM MgSO_4_, 1.5 mM HEPES buffer pH 7.1–7.3 and 0.6 μM methylene blue).

### Overexpression of human wild-type and mutant *ZFPM2* mRNA in zebrafish

The human wild-type and mutant *ZFPM2* sequences were cloned into the pEasy-T vector (Promega, USA). The capped and poly(A) tailed mRNA of h*ZFPM2* and its mutant were synthesized in vitro by transcribing with T7 RNA polymerase using the mMessageMachine T7 Ultra Kit (Ambion, Cat #AM1345, USA). The embryos of the transgenic cmlc2 were as follows: eGFP was collected for the microinjection at the single-cell stage. The experimental groups included the Mut, WT and control groups. For the control group, an equal volume of solution was injected. One hundred embryos were collected from each injection group and the control group. The purified mRNA of 450 pg was injected into the embryos at the single-cell stage. A minimum of three independent experiments was performed for the wild-type and mutant ZFPM2 mRNA injections.

### RNA extraction and quantitative real-time PCR

The embryos of the transgenic cmlc2 were as follows: eGFP and embryos with human wild-type or mutant ZFPM2 mRNA injections were collected 48 h post fertilization (hpf). The total RNA was extracted from the embryos at 48 hpf using the TRIzol reagent (Invitrogen, CA) and converted to cDNA using the PrimeScript RT Reagent Kit (Takara Bio, Japan). The RT-PCR reactions were performed with the SYBR Premix Ex Taq™ (Takara Bio, Japan) on the Roche 480 plus system. The real-time primers for the amplification were as follows:

- human *ZFPM2*-F: 5′-GCAAGGAGTGGAAGACAACA-3′,
- human *ZFPM2*-R: 5′-AGCTCTTCACCCTCAGAGAT-3′;
- zebrafish *nppa*-F: 5′-ACACGTTGAGCAGACACAG-3′,
- zebrafish *nppa*-R: 5′-CTCTCTGATGCCTCTTCTGTTG-3′,
- zebrafish β-actin-F: 5′-CGAGCTGTCTTCCCATCCA-3′,
- zebrafish β-actin-R: 5′- TCACCAACGTAGCTGTCTTTCTG-3′. Gene expression was normalized to β-actin and was analyzed using the relative quantification method (2^−ΔΔCt^). Three independent experiments were performed.

### Analysis of zebrafish cardiac morphogenesis

All surviving embryos at 48 h post injection were collected from each group by manually stripping the fertilization membrane under the microscope. For each group, the cardiac morphogenesis of fifty embryos at 48 h post injection was carefully observed. To analyze the heart lopping and chamber formation, the size and structure of the atrium and ventricle and their positions were compared among the groups under a Leica M205C inverted microscope. The number of embryos with cardiac defects in each group was counted. A minimum of three independent experiments was performed.

### Statistical analysis

The statistical analysis was performed using paired two-tailed Student’s *t* test (GraphPad Software, San Diego, CA) or Mantel–Haenszel Chi square test (Biostatistics Services, IUSM).

## Results

### Variants in multiple genes were found in single patients

Rare variants of key cardiac transcriptional factors and *JAG1* were identified in the patients, and detailed pathogenic prediction information is shown in Table 2. Interestingly, there are four patients, who carry multiple gene variants. Patient B393, with an ECHO diagnosis of TOF, carried the *JAG1*(c.3038A>T, p.H1013L) and *GATA6*(c.972C>G, p.H324Q) variants; patient B294, with TOF, carried the *JAG1*(c.2906T>C, p.M969T) and *GATA5*(c.274G>T, p. A92S) variants; and patients B445 and B548, both of which were diagnosed with TOF and PFO, carried two *GATA4* variants (c.1220C>A, p.P407Q;c.1138G>A, p.V380 M).

**Table 2:** Information regarding the **r**are variants identified in the TOF patients

Gene	Nucleotide change	Amino acid change	Patient ID	Diagnosis	Scores of SIFT/PolyPhen/MutationTaster	SIFT/PolyPhen/MutationTaster	ExAC/1 KG (frequency)	Patients (n = 106)	Internal database	ClinVar
*JAG1*	c. 1511A>G	N504S	B151	TOF/PFO	0.04/0.07/0.1	D/B/D	0.00004/0	1	2	Likely pathogenic
	c. 3038A>T	H1013L	B393*	TOF	0.02/0.601/1	D/P/D	0.0001/0	1	0	
	c. 2906T>C	M969T	B294*	TOF	0.042/0.001/1	T/B/D	0/0	1	0	
	c. 806C>G	P269R	B431	TOF/ASD	0/1/1	D/D/D	0/0	1	0	
*GATA4*	c. 1220C>A	P407Q	B445,B548*	TOF/PFO	0.05/0.145/1	D/B/D	0.0006/0.0012	2	1	Pathogenic
	c. 1138G>A	V380 M	B445,B548*	TOF/PFO	0.49/0.002/0.002	T/B/N	0.0063/0.0156	2	2	
*GATA5*	c. 943T>A	S315T	B314	TOF/PFO	0.69/0.006/0	T/B/N	0/0	1	0	
	c. 274G>T	A92S	B294*	TOF	0.83/0.097/0.001	T/B/N	0/0	1	0	
*GATA6*	c. 331G>A	D111 N	B413	TOF	0.19/0.069/1	T/B/D	0/0	1	0	
	c. 972C>G	H324Q	B393*	TOF	0.3/0.846/0.026	T/P/N	0/0	1	0	
*ZFPM2*	c. 3442G>A	E1148 K	B430	TOF/PFO	0/0.985/1	D/D/D	0/0	1	0	
	c. 3014A>G	E1005G	B546	TOF	0.2/0.073/1	T/B/D	0.0001/0.0002	1	0	
*TBX2*	c. 2139dupG	Frameshift	B326	TOF/AVSD	././.	././.	0/0	1	0	
*TBX5*	c. 409G>T	V137L	A1114	TOF/PDA/PFO	0.02/0.772/1	D/P/D	0/0	1	0	
*CITED2*	c.-1A>T	Splice	B303	TOF/PAA	././1	././D	0/0	1	0	

* More than one rare variant was found in one case

*TOF* Tetralogy of Fallot, *ASD* Atrial septum defect, *ACSD* Atrioventricular septum defect, *LSVC* Left superior vena cava, *PDA* Patent ductus arteriosus, *PFO* Patent foramen ovale, *PAA* pulmonary artery absent, SIFT, “D” meaning deleterious, score less than 0.05, “T” meaning tolerated, score greater than or equal to 0.05; PolyPhen2, “D” meaning likely damaging, 0.957 ≤ score ≤ 1, “P” meaning likely damaging, 0.453 ≤ score ≤ 0.956, “B” meaning benign, 0 ≤ score ≤ 0.452; MutationTaster, “A” represents as disease causing automatic meaning known deleterious reported in HGMD/ClinVar/dbSNP, “D” represents as disease causing meaning likely deleterious, “N” represents polymorphism or likely harmless, “P” represents polymorphism automatic meaning known harmless. ExAC, Exome Aggregation Consortium; 1 KG, 1000 genome; Internal database: n = 3215, whole- exome sequencing data from the molecular laboratory of the Children Hospital of Fudan University; “0” in frequency means didn’t find in the database

### Rare variants in the *GATA* family member genes

**Fig. 1:**
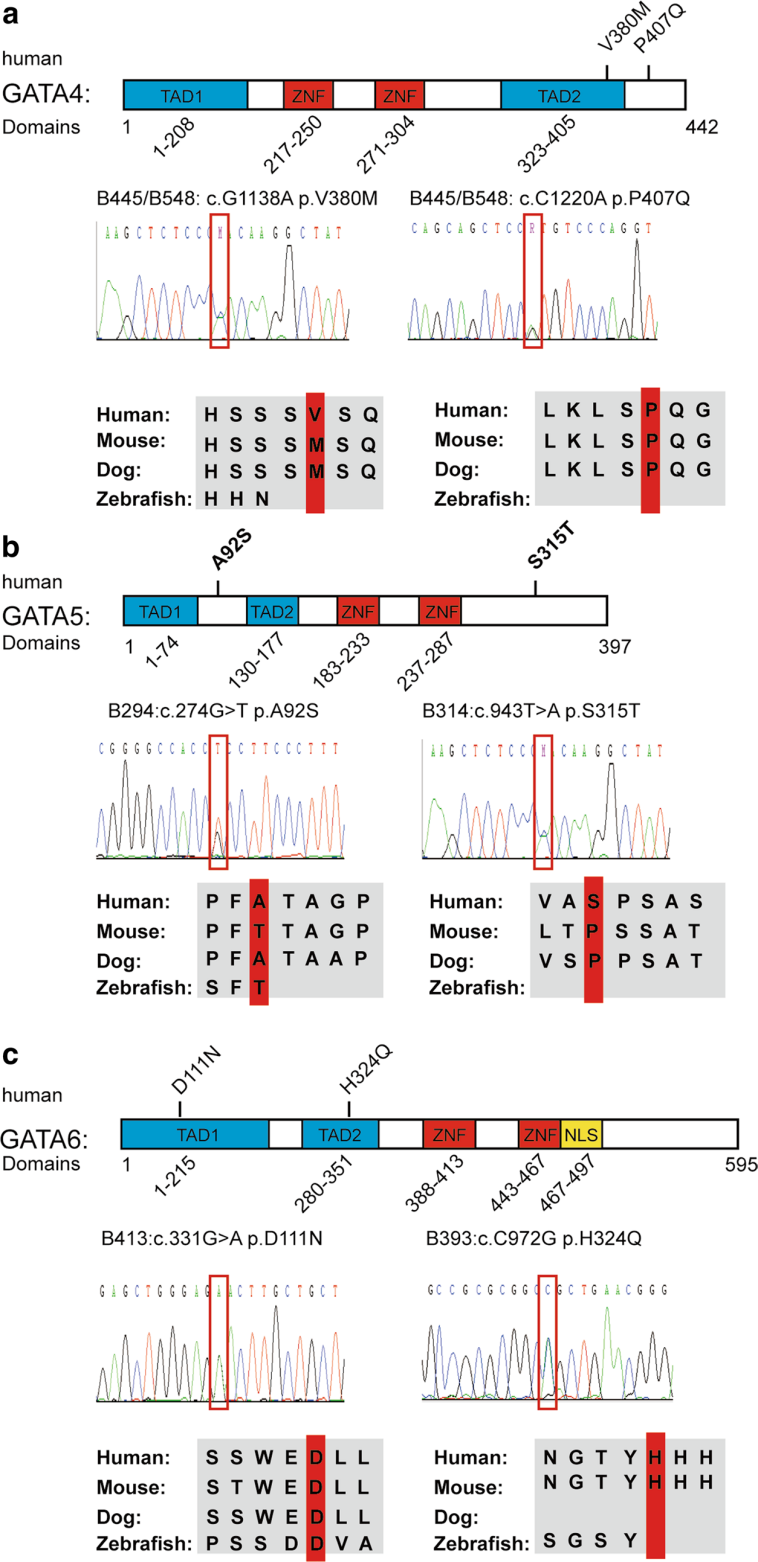
Variants in the *GATA* family members. *Blue boxes* represent the transcription activation domains (TADs), and *red boxes* indicate the Zinc finger domains (ZFN). Sanger sequencing data are displayed, and the variants are shown in the *red frames*. Alignment of amino acid residues adjacent to the variants shows a conservation among different species, including human, mouse, dog and zebrafish. **a** Human GATA4 protein domain with the variants identified in the TOF patients. The GATA4 protein is composed of four functional domains. V380 M is located in the second TAD, and P407Q is located near the second TAD. Sanger sequencing data are displayed, and the variants are shown in the *red frames*. Amino acid in 407 is conserved in human, mouse and dog but not in zebrafish. **b** Human GATA5 protein domain with the variants identified in the TOF patients. GATA5 protein also has four functional domains, and the two novel variants are not located in the functional domain. Sanger sequencing data are displayed, and the variants are shown in the *red frames*. **c** Human GATA6 protein domain with the variants identified in the TOF patients. The GATA6 protein has five functional domains, including two TADs, two ZNFs and one nuclear location signal (NLS), which is presented by the *yellow box*. The variants D111 N and H324Q are localized at the TAD1 and TAD2, respectively. The amino acid in 111 is highly conserved in human, mouse, dog and zebrafish

Two variants of *GATA4* (c.1220C>A, p.P407Q; c.1138G>A, p.V380 M) were found in both patients B445 and B548. These patients were diagnosed with TOF and PFO. Variant V380M was located in the second transcription activation TAD domain (TAD2) of GATA4 (Fig. 1a). P407Q was reported to be pathogenic in the human genome mutation database (HGMD) and ClinVar. This variant is conserved (Fig. 1a). The frequencies of the two variants are approximately 2% in these patients (Table 2).

Two novel variants of *GATA5* (c. 943T>A, p.S315T and c. 274G>T, p.A92S) were identified. The frequencies were both approximately 1% (Table 2). They were located in the N-terminal and C-terminal of GATA5 in addition to the functional domains (Fig. 1b).

As for *GATA6*, two novel variants (c.331G>A, p.D111N and c.972C>G, p.H324Q) were detected. The frequencies in this cohort were both approximately 1% (Table 2). Furthermore, the two variants were all located in the TAD domains (Fig. 1c). The variant (c.331G>A, p.D111N) is more conserved in different species.

### The novel variants of *ZFPM2* attenuated the transcriptional activation of GATA4

**Fig. 2:**
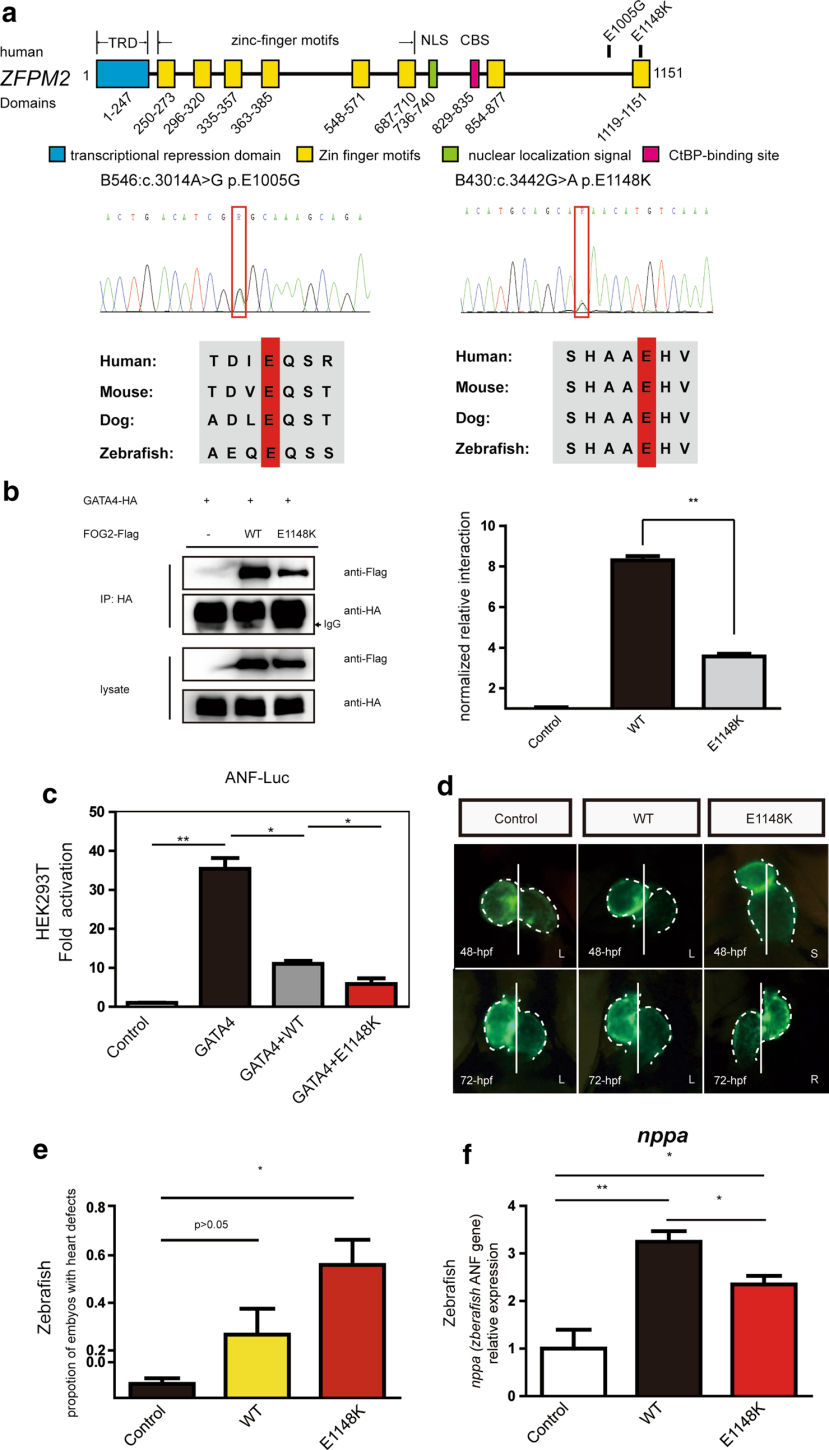
The variant of *ZFPM2* attenuated the transcriptional activation of GATA4 and contributed to the cardiac abnormalities in zebrafish. **a** Diagram of the human ZFPM2/FOG2 protein domain with the location of its variants identified. The eight zinc-finger motifs (ZNF) are represented by *yellow boxes*. The nuclear localization signal (NLS) is indicated by a *green box*. The putative CtBP-binding site (CBS) is represented by a *pink box*. The N-terminal transcriptional repression domain (TRD) is indicated by a *blue box*. E1148 K found in B430 is located at the eighth Zinc-finger domain, and E1005G found in B548 is located in the seventh and eighth domains. Sanger sequencing shows the variant in the *red frame*. Both are conserved in human, mouse, dog and zebrafish. The amino acid residue altered by the mutation is shown in the *red box*. **b** Co-immunoprecipitation assays in HEK293T cells revealed that the E1148 K variant significantly damaged the interaction between ZFPM2 and GATA4 on the western blot. The semi-quantitative analysis of the western blot results shows that the E1148 K mutant ZFPM2 protein significantly disrupted the interaction with GATA4 compared to the wild-type ZFPM2 protein. The experiment was repeated three times. (p < 0.05 *, p < 0.01 **) **c** Luciferase reported gene assays were performed. The result revealed that the wild-type ZFPM2 protein could inhibit the transcriptional activation of GATA4 on the promoter of ANF. Furthermore, E1148 K inhibited the transcriptional activation of the GATA4 on the ANF promoter significantly more than the WT. Experiments were performed in triplicate, and the mean and standard deviations are shown. (*p < 0.05 and **p < 0.01). **d** Overexpression of the mutant ZFPM2 protein (E1148 K) contributes to the abnormal cardiac morphogenesis in the zebrafish embryos. In the control and wild-type human ZFPM2 mRNA injection groups, the embryos show normal cardiac left–right asymmetry at 48 and 72 hpf. The normal cardiac left–right asymmetry is “left (L)”: the ventricle is on the left side of the midline, and the atrium is on the right side. However, in the mutant mRNA injection group, the embryos showed the “right (R)” or “straight (S)” abnormal left–right asymmetry. **e** In each group, the cardiac morphogenesis of fifty embryos (n = 50) was analyzed. The number of embryos with cardiac defects, including size, left–right asymmetry, looping and pericardialites, was counted. The proportion of embryos with cardiac defects in the mutant human ZFPM2 protein (E1148 K) injection group is significantly higher than that in the other two groups. Three independent experiments were performed. (*p < 0.05 and **p < 0.01). **f** The *nppa* (zebrafish ANF gene) mRNA level in the embryos that received the E1148 K mutant human ZFPM2 mRNA injection is lower than that in the wild-type human ZFPM2 mRNA injection group. The mean and standard deviations are shown. Experiments were performed in triplicate. (*p < 0.05 and **p < 0.01)

Two missense variants (c.3442G>A, p.E1148K; c.3014A>G, p.E1005G) of *ZFPM2* were identified in patient B430 and B546, respectively (Table 2). The novel E1148K variant was located at the eighth Zinc-finger domain. This variant is conserved in different species (Fig. 2a). We speculated that this variant may damage the interaction with GATA4. Co-immunoprecipitation assays in HEK293T cells were performed. Interestingly, the western blotting results revealed that the variant significantly damaged the interaction between the two proteins. Based on the semi-quantitative analysis, the interaction between the E1148K mutant ZFPM2/FOG2 with GATA4 was decreased by 50% compared to that with the wild-type ZFPM2 protein (Fig. 2b). Thus, this result prompted us to investigate whether the changes in the interaction affect the transcription activity of GATA4 on the promoter of the target gene. The luciferase reporter gene assays revealed that the wild-type ZFPM2 protein could inhibit the transcriptional activation of GATA4 on the promoter of ANF. Furthermore, the E1148K mutant ZFPM2 protein displayed a significantly greater inhibition of the transcriptional activation of GATA4 than the wild-type protein (Fig. 2c).

The in vitro study revealed that the novel variants of *ZFPM2* attenuated the transcription activity of GATA4. Then, we attempted to determine whether the variant could be deleterious to cardiac development in vivo. Therefore, wild-type mRNA and E1148K mutant mRNA of ZFPM2 were injected into zebrafish embryos. At 48-hpf, in control and wild type mRNA injection groups, the chambers of the ventricle and atrium had been formed. In the ventral view, the ventricle and atrium were located at the left and right sides of the midline, respectively. The cardiac left–right asymmetry is normal. However, in the mutant mRNA injection group, the number of embryos with an abnormal cardiac chamber structure and left–right asymmetry was increased. Moreover, the ventricle and atrium were located at the midline rather than on the left and right sides, which is similar to the phenotypes of heterotaxy (Fig. 2d). Furthermore, the ventricle and atrium of a few embryos displayed the complete mirror image of normal, which is similar to the phenotypes of situs inversus. By 72-hpf, the defects of the chamber and left–right asymmetry became obvious. In the E1148K mutant mRNA group, 5% of the embryos had severe defects, such as eye defects and spinal curvature. However, there were no embryos with severe defects in the control or wild-type mRNA injection groups. In total, in the E1148K mutant mRNA injection group, 56% of the embryos showed cardiac defects. However, the embryos with cardiac defects accounted for only 6 and 27% in the control and wild-type mRNA injection groups, respectively (Fig. 2e). Notably, the expression of *nppa* (zebrafish ANF gene) in the embryos with the wild-type and E1148K mutant ZFPM2 overexpression was significantly increased compared with that in the control embryos. Furthermore, the expression in the E1148K mutant group is significantly lower than that in the wild-type group, which is consistent with the in vitro result (Fig. 2f).

### Rare variants of *JAG1* were identified

**Fig. 3:**
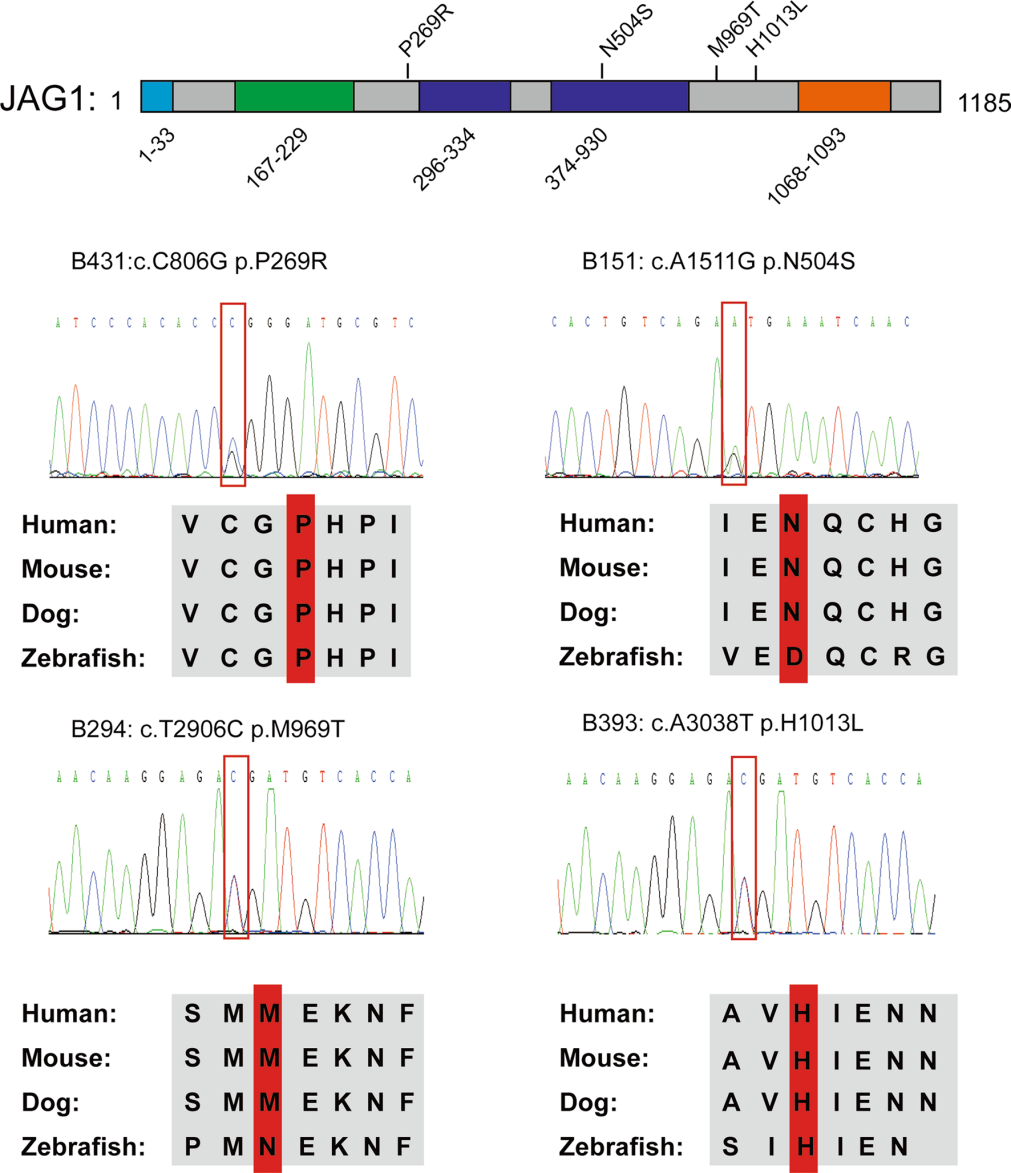
Rare variants of *JAG1* identified in patients. Human JAG1 protein structures with the location of the variants identified are shown above the diagram. The amino acid positions of the putative JAG1 functional domains are shown below the protein diagram. *Blue box* represents the signal peptide, and the *green box* indicates the DSL domain. In addition, the *dark blue boxes* show the EGF repeat domains, and the *orange box* represents the transmembrane domain. Four variants (p.P269R, p.N504S, p. M969T, p. H1013L) of JAG1 were identified and were conservative in different species, including human, mouse, dog and zebrafish. The amino acid residue altered by the mutation is shown in the *red frame*. All variants were confirmed by Sanger sequencing and are shown in the *red frames*. The variant p. N504S is located on the EGF repeats domain, and the other three variants are not in the functional domain

Four rare variants of *JAG1* were identified in the four independent patients as follows: B151: N504S; B393: H1013L; B294: M969T; and B431: P269R. The N504S variant is located in the EGF repeat domain (Fig. 3). All variants of *JAG1* were conserved in different species. These four patients were diagnosed with TOF. Additionally, patient B294 and B393 also carried GATA family member gene mutations.

### Other cardiac transcriptional factor variants

In addition, novel variants were detected in the TBX family members and *CITED2* in three individuals with TOF. The variant of *TBX2* (c.2139dupG) was found in a patient with TOF and AVSD; variant *TBX5* (c.409G>T, p.V137L) was found in a TOF patient with PDA and PFO; and the *CITED2* (c.-1AT) variant was found in a patient with TOF and PAA (Table 2). These variants are novel, and there is no record in ExAC, 1000 genome, or our internal database.

## Discussion

During the past decade, a number of pathogenic mutations in different genes have been identified in CHD by Sanger sequencing of single genes, TES, or whole-exome sequencing, and new candidate genes and variants were constantly reported in CHD patients [30–33]. In this study, using TES, we designed a twenty-nine gene panel to sequence the coding regions of these candidate genes in 106 sporadic TOF patients. We found potential pathogenic variants in the GATA family member genes, ZFPM2 and the JAG1 gene. In addition, novel variants in the TBX family members and CITED2 have been identified. These results suggest that TES is an efficient method for next generation sequencing (NGS) based custom designed capture panels.

The GATA family members GATA4, GATA6 and GATA5 are reported to be associated with CHD. These genes are characterized by transcriptional activation domains and zinc-finger domains, which are crucial for the transcription of cardiac genes. A deficiency in these genes in mice contributed to cardiac abnormalities [34–36]. Mutations in these genes were identified in patients with various types of CHD. In this study, two variants were identified in TOF patients. P407Q was identified in CHD patients in a previous study [37]. P407Q is recorded in HGMD and ClinVar as a pathogenic variant. In addition, the V380 M variant was identified in a VSD patient in a previous study [18]. Moreover, two novel variants were identified in the TAD domains of GATA6, both of which are novel variants.

In this study, rare variants of *ZFPM2* were identified in patients B430 and B546. Mutations in *ZFPM2* were previously identified in patients with TOF and DORV [22, 23]. ZFPM2 (FOG2), which is a transcriptional regulation factor, is reported to show an expression pattern that is consistent with that of GATA family members in cardiac embryogenesis. Moreover, ZFPM2can interact with the GATA family members via the zinc finger domain of GATA to regulate the activities of those members [38]. The E1148K variant was novel and predicted to be pathogenic. This variant was located at the eighth Zinc-finger domain of the *ZFPM2* gene, and we speculated that this variant may damage the interaction with GATA4. Interestingly, the co-immunoprecipitation assay results obtained by western blotting revealed that the variant significantly damaged the interaction between the ZFPM2 protein and GATA4. During different stages of development, FOG2 can combine with GATA4 to activate or repress gene expression [39]. The atrial natriuretic factor (ANF) was reported to be the target gene of GATA4 [40]. The transcription of ANF could be activated by GATA4 on the promoter. When FOG2 combines with GATA4, it can inhibit the transcription activation role of GATA4 on the promoter of ANF. However, FOG2 alone can display a moderate transcription activation role on the promoter of ANF [39]. To further confirm the effects of this variant, we compared the transcription activity of ANF between the wild-type ZFPM2 and the E1148K mutant ZFPM2 protein. Notably, the E1148K mutant ZFPM2 showed a significantly greater inhibitory role in the transcriptional activation of GATA4. To the best of our knowledge, this is the first report to demonstrate that the variant in the eighth zinc finger domain of ZFPM2 affects the interaction with GATA4 and the transcriptional activation of GATA4. Then, zebrafish were used for a functional analysis of this variant in vivo. The overexpression of mutant mRNA in zebrafish embryos resulted in cardiac morphological abnormalities. Interestingly, embryos that received the E1148K mutant mRNA injection displayed an obvious loss of the left–right asymmetry. Furthermore, we found that the overexpression of ZFPM2 could increase the expression level of *nppa* (zebrafish ANF gene) in zebrafish. Notably, the expression level of *nppa* in the embryos that received the mutant mRNA injection is significantly lower than that in the embryos that received the wild-type mRNA injection, which is consistent with the in vitro result. The experiments in the zebrafish provided strong evidence that the novel variant of ZFPM2 is pathogenic in CHD.

**Fig. 4:**
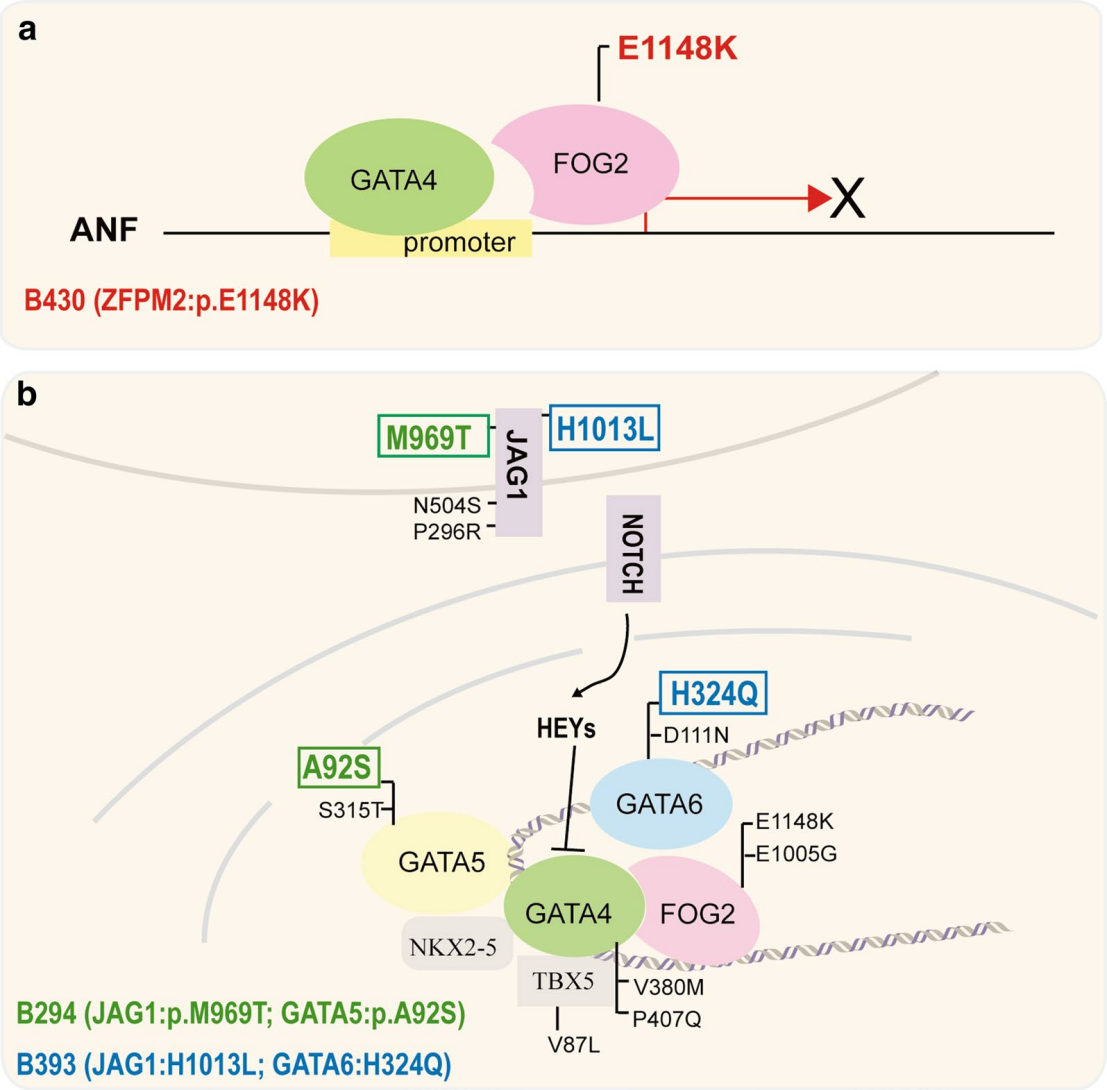
Two possible genetic etiologies of CHD. **a** Monogenic mutations in a key cardiac regulator may be the cause of CHD. A pathogenic E1148 K variant of ZFPM2 in patient B430 decreased the binding of ZFPM2 and GATA4 and attenuated the transcriptional activation of GATA4 on the promoter of ANF. **b** The majority of the variants in the GATA family member genes and JAG1 occurred sporadically in the patients. However, patients B294 and B393 carried two variants in two genes. B393 carried one variant in JAG1 and one variant in GATA6 variant, shown in a *blue color*, and B294 carried one variant in JAG1 and one variant in GATA5, shown in a *green color*. The results support the notion that rare, moderate-effect gene variants occur simultaneously in patients and may increase the susceptibility to cardiac malformations. The interaction between NOTCH signaling and the GATA family members is indispensable for cardiac development

Intriguingly, multiple gene variants in *JAG1* and the *GATA* family members were detected. JAG1 is a receptor of NOTCH1, which could help the transportation of NOTCH1 to activate the genes that are downstream in the Notch signaling pathway [41]. Heterozygous mutations in *JAG1* contributed to the pathogenesis of Alagille syndrome (AGS) [42], TOF, and peripheral pulmonary stenosis (PPS) [10]. However, heterozygous *Jag1* mutant mice only showed an eye defect [43]. However, for mice that were compound heterozygous for the *Jag1* null allele and a *Notch2* hypomorphic allele showed defects that were similar to AGS, including cardiac defects [44]. These results suggested that the pathogenesis of CHD may involve multiple gene deficiencies. HEY transcriptional factors, which are activated by NOTCH signaling, could repress gene expression [45]. Previous studies showed that HEY proteins can not only interact with the GATA family members at the GATA binding DNA element but can also regulate the transcriptional activity of GATA to repress the downstream genes, such as ANF [25]. It was suggested that the interaction between NOTCH signaling and the GATA family members is indispensable for cardiac development. Therefore, in patients B393 and B294, it is likely that the accumulation of multiple gene variations could cooperatively contribute to the pathogenesis of CHD, and the heterogeneity of the CHD subtype may be related to the different genetic deficiency patterns. As described in Fig. 4, we speculate that the variants of *JAG1* likely attenuated the transcriptional activation of HEY proteins via an abnormal signaling transduction and then affected the transcriptional regulation of the GATA family members, which might have an additive effect for the variants in these members.

The TBX transcription factors share a highly conserved DNA-binding domain, play vital roles in embryonic development and are required for cardiac morphogenesis in mammals [46]. A novel c.2139dupG variant in *TBX2* was found in a patient with TOF and AVSD. The TBX2 gene is expressed in myocardiac tissue and the outflow tract and plays a vital role in heart chamber formation. Genomic deletions and duplications of the TBX2 gene have been associated with cardiac defects [47]. The variant of TBX5 (c. 409G>T, p. V137L) is located at the T-Box domain, which likely affects the function of TBX5.

## Conclusions

In summary, we identified rare variants in cardiac transcription factors and *JAG1* in sporadic patients with TOF. Notably, a novel pathogenic variant of *ZFPM2* was proven to attenuate the interaction between ZFPM2 with GATA4 and inhibit the transcription activity of GATA4. In addition, the overexpression of E1148 K mutant mRNA in zebrafish embryos resulted in cardiac malformation in the zebrafish. Furthermore, variants in the *GATA* family member genes and *JAG1* were identified. This result suggested that multiple gene deficiencies could contribute to the pathogenesis of CHD. We believe that with the development of genetic modification tools, such as the CRISPR-Cas9 system, which could provide more support for single-gene or multigene functional studies, better knowledge regarding the genetic etiology of CHD will be established in the future.

## Additional files

**Additional file 1: Table S1.** Patients’ clinical characteristics.

**Additional file 2: Table S2.** Information of twenty-nine candidate genes.

## Abbreviations

CHD: congenital heart diseases
TOF: Tetralogy of Fallot
OMIM: online mendelian inheritance
NGS: next-generation sequencing
WES: whole exome sequencing
TAD: transcriptional activation domains
NuRD: nucleosome remodeling and deacetylation
TES: target exome sequencing
SNVs: single-nucleotide variations
ANF: atrial natriuretic factor
cmlc2: eGFP: cardiac myosin light chain 2:eGFP reporter
hpf: hour post fertilization
HGMD: human genome mutation database
AGS: Alagille syndrome
PPS: peripheral pulmonary stenosis
PDA: patent ductus arteriosus
PFO: patent foramen ovale
PAA: pulmonary artery absent
