# Supplementary material for: Multiple gene variations contributed to congenital heart disease via GATA family transcriptional regulation
Source: J Transl Med. 2017 Apr 3;15:69. doi: 10.1186/s12967-017-1173-0 (PMC5379520; doi:10.1186/s12967-017-1173-0)
Supplement: Supplementary file 2 — Additional file 2: Table S2. Information of twenty-nine candidate genes. [file 12967_2017_1173_MOESM2_ESM.docx]

Table S2: Information of twenty nine candidate genes.

| GENE NAME | NM | Mutations_No.  ( till 2016.03) | CHD Phenotype |
| --- | --- | --- | --- |
| *ALDH1A2* | [NM_003888.3](http://www.ncbi.nlm.nih.gov/entrez/viewer.fcgi?val=NM_003888.3) | 2 | TOF |
| *CFC1* | NM_032545.3 | 15 | CHD/Dextro-looped TGA/TGA |
| *CITED2* | NM_006079 | 11 | CHD |
| *CRELD1* | NM_015513 | 11 | AVSD/AVCD/CHD |
| *FGF10* | NM_004465 | 1 | TOF |
| *FOXH1* | NM_003923 | 32 | CHD/VSD |
| *GATA4* | NM_002052 | 140 | CHD/VSD/TOF/AVSD… |
| *GATA5* | NM_080473 | 27 | BAV/TOF/ASD… |
| *GATA6* | NM_005257 | 29 | TOF/CHD/VSD… |
| *GDF1* | NM_001492 | 11 | CHD/RAI |
| *GJA5* | NM_005266 | 34 | TOF/CHD/TGA… |
| *HAND1* | NM_004821 | 6 | Cardiac malformations/VSD |
| *HAND2* | NM_021973 | 5 | CHD/TOF/VSD |
| *IRX4* | NM_016358 | 3 | CHD |
| *JAG1* | NM_000214 | 10 | TOF |
| *NEXN* | NM_144573 | 15 | ASD/Cardiomyopathy, dilated… |
| *NKX2-5* | NM_004387 | 94 | CHD/ASD/VSD/TOF… |
| *NKX2-6* | NM_001136271 | 7 | CHD/TOF/VSD… |
| *NODAL* | NM_018055 | 7 | TOF/CHD/TGA |
| *PITX2* | NM_000325 | 10 | ARS/Iridogonio dysgenesis… |
| *TBX1* | NM_080647 | 53 | CHD/TOF/VSD… |
| *TBX2* | NM_005994 | 5 | VSD/Complex heart defect & skeletal abnormalities |
| *TBX20* | NM_001077653 | 21 | VSD/TOF/ASD… |
| *TBX5* | NM_000192 | 112 | Holt-Oram syndrome/CHD/BAV… |
| *TFAP2B* | NM_003221 | 12 | Char syndrome/PDA/Cardiac defects… |
| *TLL1* | NM_012464 | 3 | ASD |
| *WDR5* | NM_052821 | 1 | CHD |
| *ZFPM2* | NM_012082 | 18 | DORV/TOF/ASD… |
| *ZIC3* | NM_003413 | 22 | Cardiac malformation/CHD |

Transposition of the great arteries, TGA; Atrioventricular canal defect, AVCD; Bicuspid aortic valve, BAV; Right atrial isomerism, RAI; Axenfeld-Rieger syndrome, ARS; Patent ductus arteriosus, PDA; Double outlet right ventricle, DORV
